# On the effect of β phase on the microstructure and mechanical properties of friction stir welded commercial brass alloys

**DOI:** 10.1016/j.dib.2015.11.013

**Published:** 2015-11-21

**Authors:** Akbar Heidarzadeh, Tohid Saeid

**Affiliations:** Faculty of Materials Engineering, Sahand University of Technology, Tabriz, Iran

**Keywords:** Friction stir welding, Brass, Microstructure, Mechanical properties

## Abstract

Conventional fusion welding of brass (Cu–Zn) alloys has some difficulties such as evaporation of Zn, toxic behavior of Zn vapor, solidification cracking, distortion, and oxidation [1], [2], [3]. Fortunately, friction stir welding (FSW) has been proved to be a good candidate for joining the brass alloys, which can overcome the fusion welding short comes [4], [5], [6], [7]. The data presented here relates to FSW of the single and double phase brass alloys. The data is the microstructure and mechanical properties of the base metals and joints.

**Specifications Table**

**Table:** 

Subject area	*Materials Science and Engineering*

More specific subject area	*Friction Stir Welding and Processing*
Type of data	*Table, images*
How data was acquired	*Table was acquired using the tensile test results. The images was captured using optical microscope (OM) and scanning electron microscope (SEM)*
Data format	*Analyzed*
Experimental factors	*The composition of the plates was 37* *wt% Zn and 63* *wt% Cu. The plates were annealed before welding at 50* °*C for 1* *h. For producing the double phase alloy, the plate was heated at 810* °*C for 70* *min, and then quenched in water at room temperature. A tool with a cylindrical shoulder (12* *mm diameter) and a cylindrical pin (3* *mm diameter and 1.7* *mm length) made of H13 hot work tool steel was used. FSW was conducted at rotational speed of 450 rpm and traverse speed of 100* *mm/min.*
Experimental features	*FSW was done parallel to the initial rolling direction of the plates. The tilt angle of the tool relative to the normal direction of the plate surfaces was kept constant at 2.5*°. *The joints were produced at room temperature.*
Data source location	*Sahand University of Technology, Tabriz, Iran*
Data accessibility	*Data is with this article*

**Value of the data**

• The parameters presented here may help to obtain defect free friction stir welded joints of single and double phase brass alloys.
• Similar parameters can be used for friction stir welding of the other types of copper and brass alloys.
• Other researchers can use the presented data as a guideline in selecting the initial microstructure of the base materials to be friction stir welded.

## 1 Data

The tensile properties of the base materials and friction stir welded single and double phase alloys are presented here. In addition, the XRD pattern, OM and SEM images of the base materials and joints are the other part of the data.

## 2 Experimental design, materials, and methods

**Fig. 1:**
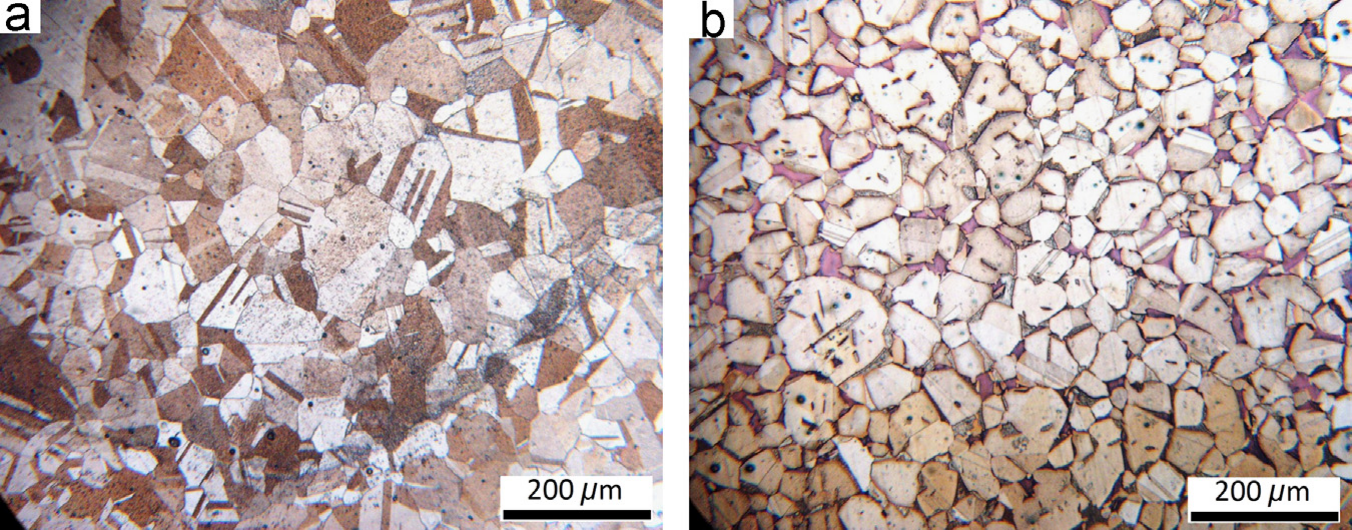
Microstructures of the base metals: (a) single phase brass, and (b) double phase brass.

**Fig. 2:**
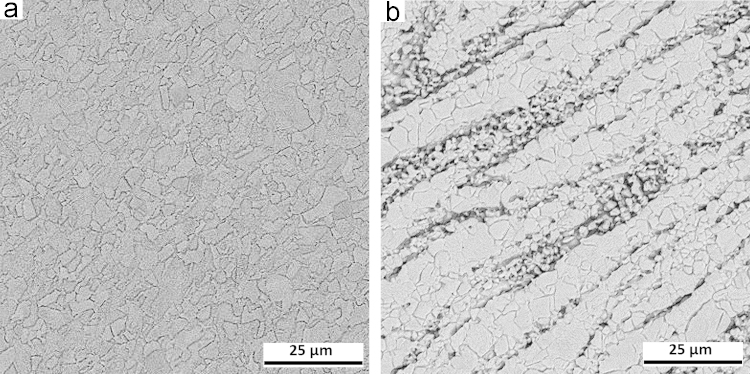
Microstructures of the joints: (a) single phase brass, and (b) double phase brass.

**Fig. 3:**
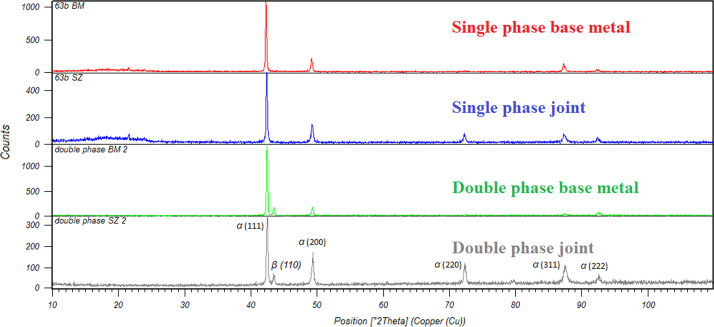
XRD patterns of the base materials and joints.

The single and double phase brass plates with dimensions of 100 mm×100 mm×2 mm were friction stir welded. After visual inspection, the microstructures of the joints were studied using optical microscope (OM) and scanning electron microscope (SEM) working with an accelerating voltage of 20 kV. The metallographic samples were cut from the joints, transverse to the welding direction, then polished and etched with a solution of 20 ml nitric acid and 10 ml acetic acid. The resulting microstructures are shown in Fig. 1, Fig. 2. X-ray diffraction (XRD) was performed to calculate the dislocation densities of the joints. The XRD patterns for the base materials and joints are presented in Fig. 3. In addition, the tensile test specimens were machined perpendicular to the welding direction with a gauge size of 12 mm (length)×3 mm (width)×2 mm (thickness). The tensile tests were conducted at a cross head speed of 1 mm/min. The tensile test data are presented in Table 1 in brief.

## Figures and Tables

**Table 1:** Tensile properties of the base materials and joints of the single and double phase brass alloys.

Tensile properties	Single phase brass		Double phase brass	
Base metal	Joint	Base metal	Joint
Ultimate tensile strength (MPa)	248	335	285	394
Elongation (%)	68	47	52	38
